# Extracellular Vesicle-Mediated Delivery of Ultrasmall Superparamagnetic Iron Oxide Nanoparticles to Mice Brain

**DOI:** 10.3389/fphar.2022.819516

**Published:** 2022-04-07

**Authors:** Naseer A. Kutchy, Rong Ma, Yutong Liu, Shilpa Buch, Guoku Hu

**Affiliations:** ^1^ Department of Pharmacology and Experimental Neuroscience, University of Nebraska Medical Center, Omaha, NE, United States; ^2^ Department of Anatomy, Physiology, and Pharmacology, School of Veterinary Medicine, St. George’s University, St. George’s, Grenada; ^3^ Department of Pharmacology, School of Basic Medicine, Tongji Medical College, Huazhong University of Science and Technology, Wuhan, China; ^4^ Department of Radiology, University of Nebraska Medical Center, Omaha, NE, United States

**Keywords:** brain drug delivery, extracellular vesicles, magnetic resonance imaging, nanoparticles, intranasal perfusion

## Abstract

Extracellular vesicles (EVs) are small lipid membrane-bound vesicles that can pass the blood–brain barrier. Therefore, EVs could be used for the delivery of therapeutics to the brain. Herein, we investigated the biodistribution of intranasal perfusion of ultrasmall superparamagnetic iron oxide (USPIO)-labeled astrocyte-derived EVs (ADEVs) in mice. We used Western blotting, transmission electron microscopy (TEM), and nanoparticle uptake assay to characterize ADEVs. In addition, intranasal perfusion coupled with magnetic resonance imaging (MRI) was employed to determine the distribution of USPIO-labeled ADEVs in mice. Our results showed the uptake of USPIO by mouse astrocytes and ADEVs. In addition, we confirmed the biodistribution of ADEVs in the brain and other internal organs, including the kidneys, liver, and spleen. Our results suggest that USPIO did not affect mouse astrocyte cell survivability and EV release. Therefore, intranasal delivery of therapeutic loaded EVs could be used for the treatment of various brain disorders.

## Introduction

Extracellular vesicles (EVs) are small lipid membrane-bound vesicles and are heterogeneous in nature. They were initially thought of as secreted debris of platelets and have attracted research interest enormously in the past decade. EVs are subdivided into several subtypes as per their biogenesis pathways, including small EVs (diameter of 40–150 nm), which are present inside multi-vesicular bodies (MVBs) and released into the extracellular space by the fusion of MVBs with the cell membrane, and large EVs (diameter of 150–2,000 nm) originating from the plasma membrane (EL Andaloussi et al., 2013; Raposo and Stoorvogel, 2013; Théry et al., 2018; Mathieu et al., 2019; Pegtel and Gould, 2019). EVs released from almost all cell types are recognized as messengers in intercellular communication using stored cargoes such as proteins, lipids, and RNA molecules, including miRNA, mRNA, and tRNA (Ratajczak et al., 2006; Valadi et al., 2007; Al-Nedawi et al., 2008; Peinado et al., 2012; EL Andaloussi et al., 2013; Chivero et al., 2021). Cells treated with nanoparticles have been shown to be generating EVs with nanoparticles in them (Busato et al., 2016). Furthermore, EVs have also been utilized successfully to deliver siRNAs in rodents (van den Boorn et al., 2011). EVs can cross the blood–brain barrier (BBB) easily; therefore, intranasal administration of EVs is considered a preferred non-invasive method for rapid delivery of EV-encapsulated drug(s) to the brain (Visweswaraiah et al., 2002; Lakhal and Wood, 2011; Zhuang et al., 2011; Grassin-Delyle et al., 2012). Manipulating EVs and their cargo *ex vivo* can thus be envisioned as an efficient means for delivery to target organs.

The labeling of EVs with ultrasmall superparamagnetic iron oxide nanoparticles (USPIO, 4–6 nm) provides an advantage in that they do not alter the morphology and physiology of cells (Busato et al., 2016). The USPIO nanoparticles are approved by the U.S. Food and Drug Administration (FDA) and the European Commission for use as MRI contrast agents (Daldrup-Link, 2017). Nanoparticles could be loaded with different compounds like paclitaxel (PTX), a microtubule-stabilizing agent and a potent antineoplastic against small-cell lung carcinoma and breast cancer (Weaver, 2014; Ganipineni et al., 2019). The distribution of the nanoparticle-loaded EVs could be traced by magnetic resonance imaging (MRI) techniques, which are non-invasive methods of visualization. MRI is emerging as a functional probe system, the stimulus-responsive MRI-monitored drug delivery system, pH-responsive release, and thermo-responsive release system (Kim et al., 2013; Gupta et al., 2016; Park et al., 2016; Zheng et al., 2018). Therefore, in this study, we generated USPIO-labeled astrocyte-derived EVs (ADEVs) and tracked their biodistribution in mice using MRI.

Intranasal drug administration is a non-invasive method of delivering therapeutic agents to the brain and spinal cord (Hanson and Frey, 2008). This method is efficient in delivering drugs to the central nervous system (CNS) and replaces the invasive delivery methods that lead to unwanted side effects (Sil et al., 2020; Lombardo et al., 2021). In intranasal perfusion, the delivery to the CNS is fast and takes minutes to pass the BBB and reach the CNS along the olfactory and trigeminal neural pathways (Hanson and Frey, 2008) (Thorne et al., 1995; Chen et al., 1998).

This study demonstrated that USPIO exposure results in labeling EVs with USPIO released from astrocytes but does not affect cellular morphology, survivability, and EV release. Furthermore, intranasal perfusion of USPIO-labeled ADEVs showed that labeled EVs were localized in the brain, kidneys, and liver, as evidenced by MRI scanning.

## Materials and Methods

### Animals

C57BL/6N wild-type mice (male, 6–8 weeks) used in this study were purchased from Charles River Laboratories, Inc. (Wilmington, MA, United States) and housed in the animal facility of the University of Nebraska Medical Center (UNMC) with free access to water/food and 12-h light/dark cycle and controlled temperature and humidity. Previous studies have demonstrated gender difference effects of intranasal delivery nanoparticle drugs in aged mice but not in young mice (Ma et al., 2016; Xu et al., 2020). This study aimed to test the concept of ADEV delivery to the brain; to reduce the variables, we used only male mice. All animal protocols were reviewed and approved by the Institutional Animal Care and Use Committee (IACUC) at the UNMC.

### Cell Cultures

The mouse astrocytic cell line C8D1A [ATCC^®^ CRL-2541™; American Type Culture Collection (ATCC), Manassas, VA, United States] was cultured as described previously (Sturdivant et al., 2016) and maintained in high-glucose Dulbecco’s modified Eagle medium (DMEM) supplemented with 10% heat-inactivated fetal bovine serum (FBS), glutamine (2 mM), penicillin (100 U/mL), and streptomycin (100 μg/ml). C8D1A cells were used within ten passages. Cells were serum-starved for 12 h, before being treated with USPIO.

Labeling of mouse astrocytes with ultrasmall superparamagnetic iron oxide nanoparticles (USPIO)

The commercial USPIO (magnetite Fe_3_O_4_; Sigma-Aldrich Co., St Louis, MO, United States; catalog #725331, particle size 4–6 nm, stock solution 5 mg Fe/mL) were used to label mouse astrocytic cells. The cells were incubated with an increasing concentration of USPIO nanoparticles (50, 100, 200, 300, 400 μg/ml) for 12 h, and USPIO were diluted in high-glucose DMEM supplemented with 10% heat-inactivated FBS, glutamine (2 mM), penicillin (100 U/mL), and streptomycin (100 μg/ml). After incubation, the cells were washed twice with PBS, trypsinized, and counted, and cell cytotoxicity of nanoparticles was determined by automated cell counters (Thermo Fisher Scientific).

### Nanoparticle Uptake Assay

Prussian blue staining was utilized to visibly assess the iron uptake by astrocytes that were treated with 100 μg/ml USPIO nanoparticles. Cells were seeded on coverslips kept in 12-well plates at a density of 1 × 10^6^ cells and incubated with USPIO at 100 μg/ml for 12 h. Cells were then fixed with a 2% solution of paraformaldehyde for 30 min at room temperature and washed gently with PBS thrice. The cells were incubated for 40 min in a solution of 1% HCl and 2% potassium ferrocyanide, washed twice with distilled water, and counterstained using Nuclear Fast Red for 15 min. The cells were then washed twice with distilled water and finally embedded in a mounting medium (Dako Mounting Medium, Agilent Technologies, Santa Clara, CA, United States). The nanoparticle internalization was investigated using a light microscope. Iron nanoparticles appeared as blue spots inside the cells, while the nucleus appeared red. The intensity was measured using Zen 3.4 (Blue edition). The background intensity (control) was subtracted from the USPIO-treated cells.

### Immunofluorescence

Cells cultured on slides or coverslips were fixed with 4% paraformaldehyde for 15 min at room temperature, followed by permeabilization with 0.3% Triton X-100 in PBS, and incubated with a blocking buffer containing 10% normal goat serum in PBS for 1 h at room temperature. The cells were then incubated with CoraLite^®^488-conjugated GFAP antibody (Proteintech, Rosemont, IL, United States) overnight at 4°C. The nuclei were labeled with DAPI. The slides were covered with a coverslip with ProLong Gold antifade reagent (Invitrogen, Carlsbad, CA, United States) and allowed to dry for 24 h at room temperature. Images were captured with a 20X objective.

### Real-Time PCR

To determine the expression of GAPDH, IL-6, and TNFα, cDNA was synthesized using a Verso cDNA kit (AB-1453/B; Thermo Fisher Scientific) according to the manufacturer’s instructions. Real-time PCR was performed using SYBR Green ROX qPCR Master Mix (QIAGEN, Valencia, CA, United States). The primers were as follows: mouse GAPDH: 5′-TGC​ACC​ACC​AAC​TGC​TTA​GC-3′ and 5′-ATG​CCA​GTG​AGC​TTC​CCG​TT-3’; IL6: 5′-CCC​AAT​TTC​CAA​TGC​TCT​CCT-3′ and 5′-CCA​CAG​TGA​GGA​ATG​TCC​ACA-3’; TNFα: 5′-CGA​ATT​CAC​TGG​AGC​CTC​GAA-3′ and 5′-TGT​GAG​GAA​GGC​TGT​GCA​TTG-3’. The comparative cycle threshold (Ct) method (2^ΔΔCt) was used to calculate the relative level of gene expression. The Ct values were normalized to GAPDH.

### Characterization of Astrocyte-Derived Extracellular Vesicles and Separation Using Size Exclusion Chromatography

**FIGURE 1:**
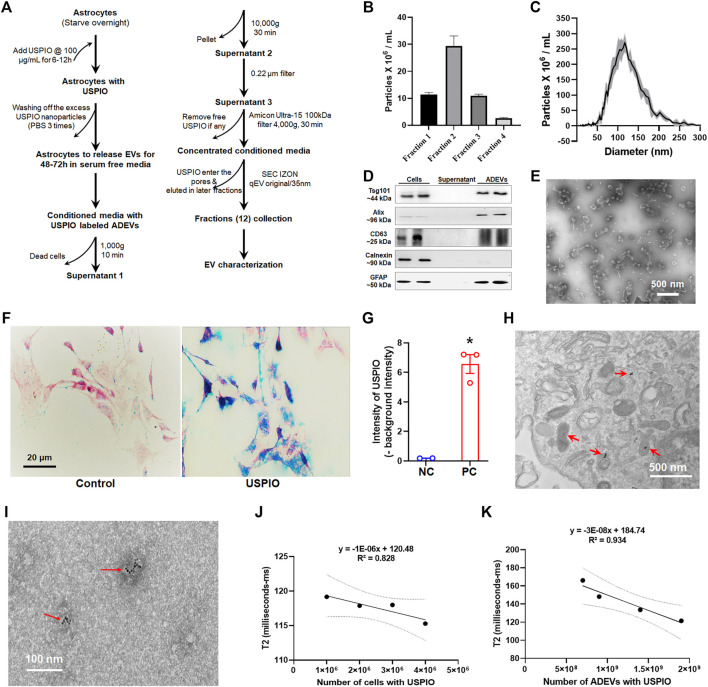
Uptake of USPIO by mouse astrocytes and characterization of EVs. **(A)** Schematic diagram of the procedure used for separating EVs from astrocyte culture by size exclusion chromatography (SEC). **(B,C)** Number and size distribution plots of EVs from selected fractions by ZetaView. **(D)** The four fractions of the qEV column with maximum EVs were pooled together for detection of EV markers using Western blotting. EV proteins were separated by sodium dodecyl sulfate–polyacrylamide gel electrophoresis (SDS-PAGE) and electroblotted onto a nitrocellulose membrane. Calnexin (non-exosomal protein) served as negative control. **(E)** TEM image of EVs isolated from mouse astrocytes via a qEV column (IZON Science). **(F)** Cells were treated with 100 μg/ml of USPIO, and uptake of USPIO nanoparticles was determined by nanoparticle uptake assay. **(G)** Blue signal intensity in (F) measured using ZEN (Blue edition) software. **(H)** TEM image of astrocytes treated with USPIO. **(I)** TEM image of EVs isolated from USPIO-treated astrocytes. **(J,K)** Decrease in T2-weighted data acquired using MRI showing a strong linear correlation between R2 values and numbers of cells (R2 = 0.828), and numbers of ADEVs (R2 = 0.934). All experiments were repeated at least three times.

EVs were isolated from serum-free conditioned medium (FBS depleted) of astrocytes using a qEV column (Izon Science, Christchurch, New Zealand). In brief, the conditioned medium was harvested, centrifuged at 1,000 X g for 10 min to eliminate cells, and again spun at 10,000 X g for 30 min. The supernatant was filtered through a 0.22-μm filter to remove cell debris and large vesicles. The collected supernatants were then concentrated using Amicon Ultra-15 100-kDa filter units and then subjected immediately to SEC on qEV original/35 nm columns (IZON Science, Christchurch, New Zealand). The columns were first rinsed with 1x filtered PBS, and 0.5 ml of supernatant was applied on top of a qEV column (columns allowed to remove all free USPIO nanoparticles, Izon Science), and twelve 0.5 ml fractions were collected (Figure 1A). Out of 12 fractions analyzed by nanoparticle tracking analysis (NTA), four EV-rich fractions (fractions 1–4), as well as 0.5 ml EV devoid supernatant of the same four fractions, were pooled and concentrated by evaporation. The EV and EV devoid supernatant samples were resuspended in the protein lysis buffer to detect EV markers—Alix, TSG101, and CD63—by Western blotting. The EV number and size distribution were analyzed using a ZetaView Particle Metrix, as previously reported (Ma et al., 2021).

### Western Blotting

Cells and EVs were lysed using a mammalian cell lysis kit (Sigma-Aldrich, St. Louis, MO, United States), as described previously (Liao et al., 2019). Proteins were separated in sodium dodecyl sulfate (SDS)–polyacrylamide gel, followed by transfer to a polyvinylidene difluoride (PVDF) membrane. The membrane was blocked with 3% non-fat dry milk and 0.05% Tween 20 in Tris-buffered saline for 1 h at room temperature. The membrane was then probed with a primary antibody in 5% non-fat milk overnight at 4°C. Primary antibodies specific for TSG101 (Proteintech; Rosemont, IL, United States), Alix (Proteintech; Rosemont, IL, United States), CD63 (Abcam, Cambridge, MA, United States), Calnexin (Proteintech, Rosemont, IL, United States), and GFAP (Abcam, Cambridge, MA, United States) were used in this study. The next day, the membrane was washed three times with TTBS for 10 min each and subsequently incubated with a secondary antibody—alkaline phosphatase-conjugated to goat anti-mouse/rabbit IgG (Jackson ImmunoResearch Laboratories, Inc., West Grove, PA, United States)—for 1 h at room temperature. The membrane was washed three times with TTBS for 10 min each and then developed using a West Chemiluminescent Substrate (Thermo Fisher Scientific, Waltham, MA, United States). All experiments were repeated at least three times, and the representative blots are presented in the figures.

### Transmission Electron Microscopy

EVs were fixed with 2% paraformaldehyde and 2% glutaraldehyde in 0.1 M PBS. Then 6 μl of EVs were gently placed on a 200-mesh formvar-coated copper grid, allowed to adsorb for 5 min, and processed for standard uranyl acetate staining. The grid was then washed with PBS three times and allowed to semi-dry for 2 min at room temperature before observation under an FEI Tecnai G2 Spirit Transmission Electron Microscope in the Electron Microscopy Core Facility at the University of Nebraska Medical Center. For the ultrastructural morphology of cells, cell pellets were fixed in 2.5% glutaraldehyde in 0.1 M cacodylate buffer for 1 h. Transmission electron microscopy (TEM) images were acquired with a Hitachi Transmission EM in the Central Electron Microscopy Facility at the University of Connecticut Health Center.

### Labeling of Ultrasmall Superparamagnetic Iron Oxide in Astrocyte-Derived Extracellular Vesicles

Astrocytes were seeded in a T75 flask at 1-2 x 10^6^ cells. When reaching 80–90% confluency, cells were treated with 100 μg/ml USPIO for 12 h. After that, the cells were washed three times with 1x PBS and incubated in a serum-free medium for 48 h. Then, the supernatant was collected and centrifuged at 1,000 X g for 10 min to eliminate cells and again spun at 10,000 X g for 30 min. The supernatant was filtered through a 0.22-µm filter to remove cell debris and large vesicles. The collected supernatants were then subjected to qEV columns (Izon Science). The columns were first rinsed with 1x filtered PBS, and 0.5 ml of supernatant was applied on top of a qEV column, and 0.5 ml fractions were collected. Out of the 12 fractions obtained from the qEV column, four fractions (1–4) having EVs with USPIO were pooled and used for intranasal perfusion. The EV number and size distribution were analyzed using the ZetaView Particle Metrix, as previously reported (Hu et al., 2013; Hu et al., 2018).

### Relaxivity of Mouse Astrocytes and Astrocyte-Derived Extracellular Vesicles

Mouse astrocytes were labeled with USPIO, as previously described, and were homogeneously distributed in a gel matrix (agarose low electroendosmosis; PanReac AppliChem; at 0.5% w/w) for *in vitro* T2 relaxation time evaluation. Cells (1 × 10^6^ to 5 × 10^6^) loaded with USPIO and USPIO-loaded ADEVs (5 × 10^8^ to 2 × 10^9^) were analyzed by MRI (Bruker BioSpec 70/20). In order to assess the *in vitro* detectability, USPIO-labeled astrocytes and ADEVs were immobilized in a gel matrix; MRI was performed using T2-weighted imaging and T2 mapping. *In vitro* T2 relaxivity (R2) was calculated from T2 mapping. ∆R_2 = R_2^EVs-R_2^Base (Eqn (EL Andaloussi et al., 2013)) was calculated on each pixel, where R_2^EVs is post-EVs administration R2, and R_2^Base is the baseline (cells without USPIO and EVs without USPIO) R2.

### Magnetic Resonance Imaging of Astrocyte-Derived Extracellular Vesicles With Ultrasmall Superparamagnetic Iron Oxide in Mouse Brain

Mice were intranasally administered with USPIO-labeled EVs (2 × 10^12^ EVs per perfusion) for 4 days, as illustrated in Table 1. MRI was performed 24 h after each EV administration. For MRI, animals were anesthetized by 1% isoflurane inhalation in a mixture of oxygen and nitrogen and were placed in an MRI system (Bruker BioSpec 70/20) on a heated bed. T2-weighted images were acquired using TurboRARE with TR/TE = 3,600/40 ms, RARE factor = 4, averaging number = 4, matrix size = 256 × 256, FOV = 20 × 20 mm^2^, 30 slices, and slice thickness = 0.5 mm T2 mapping was performed using MSME with TR = 4,600 ms, 20 echos from 7 to 140 ms with 7 ms echo spacing, matrix size = 192 × 192, FOV = 20 × 20 mm^2^, 30 slices, and slice thickness = 0.5 mm. *In vivo* T2 relaxivity (R2) was calculated from T2 mapping. Eqn (EL Andaloussi et al., 2013) was calculated on each pixel. ∆R_2 heatmaps were superimposed on anatomical (T2-weighted) images. Increased ∆R_2 values indicate EV entry into the brain. Baseline MRI of all animals before the administration of USPIO-labeled EVs was used as the respective control. The same methodology was used to take body MRI scans of the kidneys, liver, and spleen.

**TABLE 1:** Intranasal perfusion of USPIO-loaded mouse astrocyte-derived EVs.

Day	Intranasal perfusion of ADEVs and MRI scan
Day 0	• MRI pre-scan on 6 mice
	• Wait 1 h and intranasal perfusion of 2 × 10^12^ USPIO-labeled ADEVs in 50 μL PBS
Day 1	• Intranasal perfusion of 2 × 10^12^ USPIO-labeled ADEVs in 50 μL PBS
	• Wait 1 h and MRI scan
	• Intranasal perfusion of 2 × 10^12^ USPIO-labeled ADEVs in 50 μL PBS
Day 2	• Intranasal perfusion of 2 × 10^12^ USPIO-labeled ADEVs in 50 μL PBS
	• Wait 1 h and MRI scan
	• Intranasal perfusion of 2 × 10^12^ USPIO-labeled ADEVs in 50 μL PBS
Day 3	• Intranasal perfusion of 2 × 10^12^ USPIO-labeled ADEVs in 50 μL PBS
	• Wait 1 h and MRI scan
	• Intranasal perfusion of 2 × 10^12^ USPIO-labeled ADEVs in 50 μL PBS.
Day 4	• Intranasal perfusion of 2 × 10^12^ USPIO-labeled ADEVs in 50 μL PBS
	• Wait 1 h and MRI scan

### Statistical Analyses

All the data were expressed as mean ± SEM, and appropriate statistical significance was determined based on the experimental strategy using GraphPad Prism version 9.1.2. The precise statistical analyses and experimental designs, including tests performed, exact *p* values, and sample sizes, are provided in the figure legends. Non-parametric Kruskal–Wallis one-way ANOVA, followed by Dunn’s post hoc test, was used to determine the statistical significance between multiple groups, and an unpaired Student’s t test was used to compare between two groups.

## Results

### Characterization of Extracellular Vesicles Separated From Mouse Astrocytes

ADEVs were separated into 12 fractions (qEV columns allowed to separate free USPIO nanoparticles from EVs; Figure 1A). As shown in Figures 1B,C, fractions 1–4 contained maximum ADEVs, with sizes ranging from 50 to 250 nm. The ADEVs were further characterized by Western blotting for EV markers—TSG101, Alix, and CD63. As shown in Figure 1D, ADEVs were positive for all of the detected EV markers and astrocyte marker—GFAP, while the supernatants were negative for the EV markers. Calnexin (non-EV protein) served as a negative control. TEM was used to confirm the morphology and size of ADEVs further. As shown in Figure 1E, ADEVs were around 100 nm in diameter.

### Uptake of Ultrasmall Superparamagnetic Iron Oxide by Astrocytes

Astrocytes were treated with 50 μg/ml to 400 μg/ml of USPIO, followed by assessing cell survivability. As shown in Supplementary Figure S1A–D, USPIO treatment did not affect cell survivability, morphology, and response to LPS stimulation. We next sought to determine the uptake of USPIO in astrocytes using the nanoparticle uptake assay. As shown in Figure 1F, cells treated with 100 μg/ml of USPIO based on the previous study (Busato et al., 2016) showed an uptake of USPIO nanoparticles by astrocytes (Figures 1G,H).

### Relaxivity of Ultrasmall Superparamagnetic Iron Oxide-Labeled Astrocyte and Astrocyte-Derived Extracellular Vesicles

We next sought to confirm the presence of USPIO in astrocytes and ADEVs using TEM and MRI. TEM assays revealed that USPIO nanoparticles were present inside 85.7% of the cells (Figure 1H) and the presence of USPIO nanoparticles in 45% of ADEVs (Figure 1I). To evaluate the detection limit of astrocytes and ADEVs with USPIO in MRI, we used 1 × 10^6^ to 5 × 10^6^ astrocytes and 5 × 10^8^ to 2 × 10^9^ ADEVs immobilized in different gel tubes (agarose at 0.5% w/w, 1.5 ml). Unlabeled astrocytes and ADEVs, as well as free agarose gel, were used as negative controls. Relaxivity data were acquired using MRI. As T2 (transverse relaxation time) is the inverse of R2 (transverse relaxivity) and USPIO is a T2-shortening contrast agent, the lower values of T2 represent the higher levers of USPIO in the samples. As shown in Figures 1J,K, the linear correlations between R_2_ values of USPIO in astrocytes and the cell concentrations (*R* ^2^ = 0.828), and USPIO in ADEV and ADEV concentrations (*R* ^2^ = 0.934) suggest the uptake of USPIO by astrocytes and ADEVs. We also found that USPIO treatment did not significantly affect EV release (Supplementary Figure S1E).

### Magnetic Resonance Imaging of Mice Administrated With Astrocyte-Derived Extracellular Vesicles labeled with Ultrasmall Superparamagnetic Iron Oxide

**FIGURE 2:**
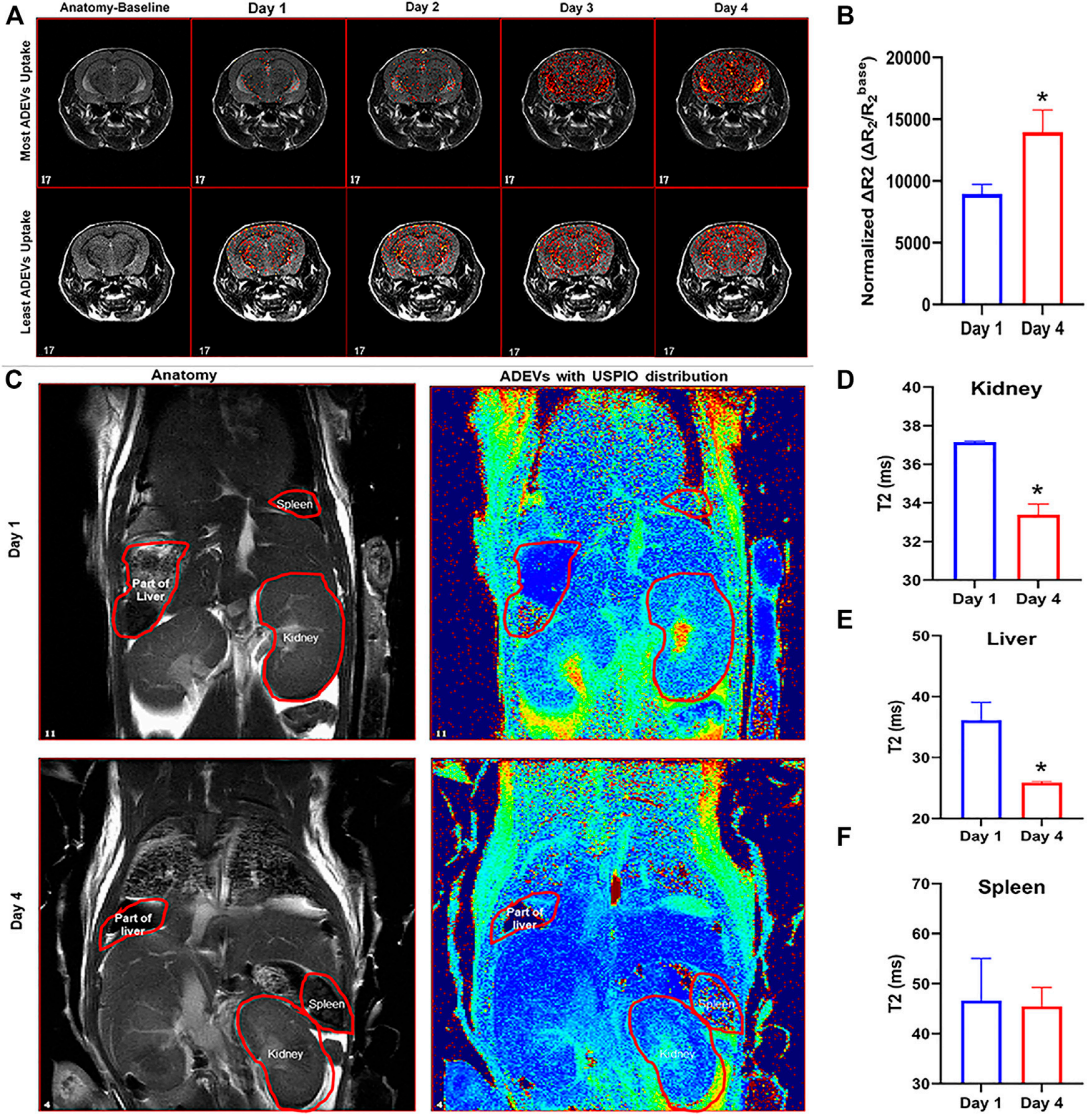
Biodistribution of USPIO-labeled ADEVs in mice. **(A)** MRI T2-weighted images showing the USPIO-labeled ADEVs were present in the brain, and their abundance increased with each intranasal perfusion. **(B)** Quantification of the USPIO-labeled ADEVs in the brain; the bar graph shows the sum of normalized ΔR2 (i.e., ΔR_2_/R_2_^base^) of all pixels in brain R_2_ maps, and value increases along with time. **(C)** MRI T2-weighted images showing the USPIO-labeled ADEVs were present in the **(D)** kidneys, **(E)** liver, and **(F)** spleen.

To examine the biodistribution of ADEVs loaded with USPIO *in vivo*, we intranasally perfused C57BL/6 mice with USPIO-labeled ADEVs. The mice were pre-scanned using MRI on day 0 to set the baseline, followed by intranasal perfusion with 2 × 10^12^ USPIO-ADEVs per mouse and another perfusion on day 1. The mice were then scanned 1 hour post-ADEV perfusion on day 1. This procedure was repeated for 4 days (Table 1). As shown in Figure 2A, ∆R_2 heatmaps were superimposed on anatomical (T2-weighted) images. USPIO-labeled ADEVs could be found in the brain 1 day after intranasal perfusion. The amount of USPIO-labeled ADEVs was increased with each intranasal perfusion in the brain (Figure 2B). Moreover, USPIO-labeled ADEVs could also be found in the kidneys (Figures 2C,D), liver (Figures 2C,E), and the spleen (Figures 2C,F).

## Discussion

This study developed a method of loading ADEVs with USPIO nanoparticles and tracked them *in vivo* using MRI. Nanoparticle uptake assay was used to determine the uptake of USPIO by mouse astrocytes without disrupting their morphology, normal growth, and release of EVs. ADEVs were separated using IZON qEV columns, followed by the characterization for EV markers by Western blotting, morphology by TEM, and number and size distribution by ZetaView. USPIO-labeled ADEVs were intranasally delivered to mice, followed by MRI scanning. MRI images suggested that USPIO-labeled ADEVs were delivered to the brain, kidneys, liver, and, to some extent, the spleen.

EVs have a pivotal role in intercellular communication under normal and diseased conditions (Patters and Kumar, 2018; Ma et al., 2021). Moreover, EVs have tremendous potential to be used as delivery vehicles for therapeutics as they can cross the BBB (Alvarez-Erviti et al., 2011; Yang et al., 2015). EVs are safer in terms of their transplantation (loss in transplanted cells), transformation to become malignant, or immune rejection (Alvarez-Erviti et al., 2011). In addition, EVs are stable and could be scaled up for therapeutics relatively easily in a stepwise process (György et al., 2015). Indeed, studies have demonstrated that delivery of EVs loaded with small RNAs such as siRNA and miRNA to rodents can reach various organs and regulate the expression of the target genes (O'Brien et al., 2020; Chivero et al., 2020; Liao et al., 2020). The current study aimed to determine the biodistribution of ADEV in live animals using a sensitive method. Herein, we successfully loaded ADEVs with USPIO nanoparticles and tracked them in mice using MRI (Busato et al., 2016). MRI results suggest that USPIO-labeled ADEVs can reach the brain, liver, and kidneys within 1 day after intranasal delivery.

Previous studies demonstrated that intranasal delivery of EVs isolated from bone marrow mesenchymal stem cells (MSCs) could suppress neurogenesis and memory dysfunction in status epilepticus mice (György et al., 2015). It has been reported that EVs were able to suppress neuroinflammation and reduce cognitive impairments in traumatic brain-injured mice (O'Brien et al., 2020). Furthermore, intranasal delivery of catalase-loaded EVs has shown significant neuroprotective effects in the Parkinson’s disease model by reducing stress-induced neuronal death (Long et al., 2017). Curcumin-encapsulated EVs could also suppress IL-6 and TNF-α expression in LPS-induced septic shock model animals (Sun et al., 2010). In line with these studies, our results suggest that USPIO-labeled EVs delivered through the intranasal route can be found in the brain, as well as the kidneys and liver.

ADEVs are endogenous brain vesicles that play a profound role in brain development, synapse formation, control of neurotransmitter release and uptake, making of trophic factors, and regulation of neuronal survivability (Ma et al., 2016; Venturini et al., 2019; Zhao et al., 2021). ADEVs thus could serve as ideal drug delivery vehicles for the treatment of brain disorders. For example, ADEVs have been used as siRNA delivery vehicles to treat LPS-mediated neuroinflammation (Liao et al., 2019). The results suggest that intranasal delivery of siRNA-loaded ADEVs significantly reversed the expression of target genes in microglia in LPS-administered mice. Efforts to engineer the ADEV for targeting specific organs and cells are ongoing and remain a major research focus. Intranasal delivery of USPIO-labeled ADEVs provides a non-invasive tool to visualize and track the engineered ADEVs in future studies.

## Conclusion

In summary, our data suggest USPIO does not show significant effects on astrocytes and the release of EVs. Our results show that USPIO can be taken up by astrocytes and released in EVs. We further demonstrated intranasal administration of ADEVs can deliver their cargo to the brain, kidneys, and liver. Therefore, engineered EVs could be harnessed to deliver therapeutics for the treatment of brain, kidney, and liver disorders.

## Data Availability

The original contributions presented in the study are included in the article/Supplementary Material, further inquiries can be directed to the corresponding author.

## Supplementary Material

Supplementary Figure S1 Effect of USPIO on cell survivability and EV release, and distribution of USPIO labeled ADEVs in the brain. **(A)** Viability of cells treated with various concentrations of USPIO. **(B)** Immunostaining for GFAP in astrocytes exposed to 100 μg/ml USPIO. **(C,D)** Real-time PCR for IL6 and TNFa in cells treated with 100 μg/ml USPIO for 12 h followed by LPS (100 ng/ml) stimulation for 6 h. **(E)** Number of EVs isolated from 100 μg/ml USPIO-treated astrocytes for 48 h. All experiments were repeated at least three times.
