# Supplementary figures and images for: Extracellular Vesicle-Mediated Delivery of Ultrasmall Superparamagnetic Iron Oxide Nanoparticles to Mice Brain
Source: Front Pharmacol. 2022 Apr 7;13:819516. doi: 10.3389/fphar.2022.819516 (PMC9022024; doi:10.3389/fphar.2022.819516)

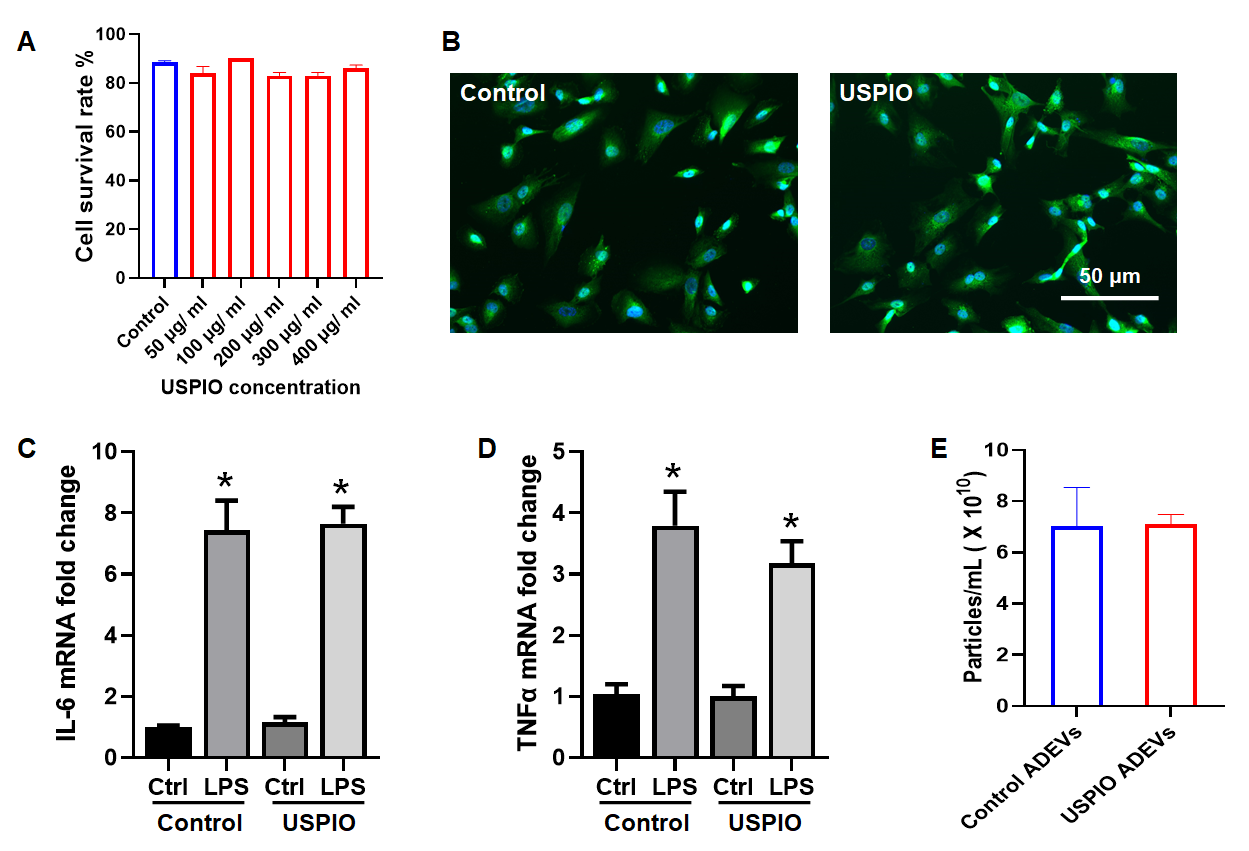

Supplement: Supplementary file 1 [file Image1.tif]
